# Genome-wide analysis of the *FAD* gene family in *Solanum tuberosum* L. reveals its involvement in cold stress tolerance

**DOI:** 10.3389/fpls.2025.1736660

**Published:** 2025-12-10

**Authors:** Xiaoyue Chu, Baoqi Yuan, Chuang Li, Xueyan Qian, Yao Yao, Jianlei Qiao, Dongquan Guo, Zhongwei Wang

**Affiliations:** 1 Jilin Agricultural University, Changchun, China; 2 Jilin Academy of Agricultural Sciences (Northeast Agricultural Research Center of China), Changchun, China

**Keywords:** cold stress, FAD gene family, lipid metabolism, phylogenetic analysis, subcellular localization, tissue-specific expression

## Abstract

Fatty acid desaturases (FADs) introduce double bonds into fatty acid chains, thereby maintaining membrane fluidity and modulating plant responses to stress. Yet the genome-wide features and functional implications of the FAD family in potato (*Solanum tuberosum* L.) remain insufficiently defined. Here, we identified 47 *StFAD* genes and analyzed their chromosomal distribution, phylogeny, conserved motifs, and promoter cis-elements. *StFAD* members were unevenly distributed across 12 chromosomes, with clusters on chromosomes 6 and 12, consistent with tandem and segmental duplications. Promoter analysis revealed abundant stress-, hormone-, and light-responsive elements, including cold-related motifs (LTR, ARE). Integrating transcriptome data with qRT–PCR, we found that *StFAD7* was strongly and persistently induced by 4 °C treatment (12–24 h). This induction coincided with attenuation of MDA accumulation after 12 h, sustained increases in SOD and CAT activities, and a transient surge of POD activity at 12 h, linking *StFAD7* expression to time-resolved physiological adjustments in tubers. Subcellular assays further showed that *StFAD7*–GFP localized predominantly to the plasma membrane, the first cellular interface challenged by low temperature, consistent with a role in lipid desaturation and redox homeostasis. These results support the hypothesis that *StFAD7* may contribute to cold-induced lipid remodeling and oxidative stress alleviation in potato. Together, they refine the landscape of the potato *FAD* family and provide a rationale for functional studies and breeding strategies aimed at improving chilling tolerance.

## 1 Introduction

Potato (*Solanum tuberosum* L.) is a major food crop cultivated in more than 150 countries worldwide, consistently ranking within the top five in global production (Camire et al., 2009; Zaheer and Akhtar, 2016). According to the FAO, global potato production reached approximately 383 million tons in 2023, with China contributing approximately 93.5 million tons, or nearly 24% of the total (Callıskan et al., 2022; Potato Pro, 2025). China has long maintained a leading position in potato cultivation area, production, and processing capacity, with its output representing more than 20% of the global total (Hu et al., 2025). Owing to its high yield potential, production efficiency, and versatility of use, the potato plays an important role in global food security, agricultural processing, and industry. Despite the large planting area, potato yield is below potential, which is further exacerbated under adverse environmental conditions such as extreme temperatures, diseases, and water stress. Among these constraints, low-temperature stress, including chilling and frost injury, represents one of the most critical abiotic factors compromising the stability of potato production.

Low-temperature stress, categorized as freezing stress (<0 °C) and chilling stress (0–15 °C), greatly limits plant growth, development, and geographic distribution (Guo et al., 2018; Ding and Yang, 2022). Freezing stress often results in ice crystal formation within cells, leading to structural disruption, severe membrane damage, and, ultimately, plant death. In contrast, chilling stress primarily alters the lipid composition of cellular membranes, which impairs membrane protein function and affects key metabolic processes such as photosynthesis and respiration, along with enzyme activity (Zhao et al., 2020; Aslam et al., 2022). At the developmental level, low temperatures can delay bud break, induce chlorosis in seedlings, reduce photosynthetic efficiency, and impair reproductive development, all contributing to yield loss (Li et al., 2022; Hou et al., 2022). Physiologically, low temperatures induce a phase transition of membranes from a liquid-crystalline to a gel state, thus reducing membrane fluidity and leading to lipid peroxidation, which is reflected by elevated malondialdehyde (MDA) accumulation (Wang et al., 2021). The concurrent excessive accumulation of reactive oxygen species (ROS) disrupts cellular macromolecules and compromises cellular integrity. Plants rely on components of the enzymatic antioxidant defense system, including superoxide dismutase (SOD) and peroxidase (POD), as well as non-enzymatic antioxidants such as ascorbic acid and glutathione, to maintain redox homeostasis under stress (Noctor et al., 2014; Wang et al., 2018; Mittler et al., 2022). At the molecular level, the ICE1–CBF–COR transcriptional cascade represents the core cold-response pathway. ICE1 activates CBF genes, which subsequently regulate a wide range of cold-inducible genes, thereby enhancing freezing and chilling tolerance (Shi et al., 2018; Verslues et al., 2023). Moreover, the abscisic acid (ABA) signaling pathway plays a critical role in stomatal regulation and cold stress adaptation. The regulatory module composed of PYR/PYL/RCAR receptors, protein phosphatase 2C (PP2C), and SNF1-related protein kinase 2 (SnRK2) has been shown to interact with the CBF–COR network, leading to the strengthening of the low-temperature response (Dou et al., 2021; Zhang et al., 2021; Wi et al., 2022). Collectively, these observations indicate that low-temperature stress impairs plant performance through membrane destabilization, ROS accumulation, and signaling pathway alterations, ultimately compromising growth and yield.

Fatty acids and their derivatives are essential components of plant cells, serving both as energy reserves and critical constituents of membrane structure and function. Fatty acid desaturases (FADs) catalyze the formation of double bonds in fatty acid chains, generating unsaturated fatty acids, and are central to the maintenance of membrane stability, the regulation of metabolism and hormone signaling, and mediating plant responses to both biotic and abiotic stresses (Dar et al., 2017; Laureano et al., 2021). The *FAD* gene family has been systematically characterized in several crops, including soybean (*Glycine max*), rice (*Oryza sativa*), peanut (*Arachis hypogaea*), tomato (*Solanum lycopersicum*), and cotton (*Gossypium hirsutum*), and *FAD* family members were found to be closely associated with stress tolerance in plants. The overexpression of soybean *GmFAD3A* and poplar *PtFAD2* significantly enhanced cold tolerance, whereas the expression of antisense *AtFAD7* of Arabidopsis reduced tolerance to drought and salt stresses (Zhou et al., 2010; Román et al., 2012). Based on subcellular localization and functional diversity, plant FAD proteins can be divided into soluble and membrane-bound types, with the latter further categorized into subfamilies such as FAD2/FAD6 (ω-6 desaturases) and FAD3/FAD7/FAD8 (ω-3 desaturases) (Cheng et al., 2022). These enzymes regulate the biosynthesis of polyunsaturated fatty acids, including linoleic acid (C18:2) and linolenic acid (C18:3), thereby modulating membrane unsaturation and phase transition temperature and consequently enhancing plant adaptation to low-temperature environments (Upchurch, 2008). Among them, FAD8, a chloroplast-localized ω-3 desaturase that is specifically induced by cold, has been shown to play a pivotal role in cold stress responses across multiple species. The overexpression of *AtFAD8* or *OsFAD8* increases C16:3 and C18:3 levels in transgenic plants and enhances their cold tolerance, whereas silencing *FAD8* reduces trienoic acid content and results in greater sensitivity to low temperatures (Román et al., 2015; Soria-García et al., 2019). Collectively, the *FAD* gene family is integral to membrane lipid remodeling and low-temperature adaptation, with *FAD8* acting as a key regulator in this process. However, although potato is particularly sensitive to chilling compared with many other crops, the contribution of *FAD*-mediated pathways to potato cold adaptation has not been systematically investigated. Furthermore, the molecular mechanisms underlying the responses of this important crop to abiotic stresses, especially cold stress, remain largely unclear.

In this study, we undertook a genome-wide search for *FAD* gene family members in potato. We characterized their chromosomal distribution, gene structure, conserved motifs, and *cis*-regulatory elements, and analyzed their evolutionary features and potential biological functions. Furthermore, we investigated the expression profiles of these genes under low-temperature stress to identify their roles in cold stress response. The findings of this study provide novel insights into the regulatory network of *FAD* genes in potato cold adaptation and offer theoretical support and valuable genetic resources for the breeding of cold-tolerant potato varieties.

## 2 Results

### 2.1 Physicochemical properties and subcellular localization prediction of StFAD proteins

The physicochemical properties of the StFAD proteins in *S. tuberosum* showed wide variation. The predicted protein lengths ranged between 106 and 764 amino acids, corresponding to molecular masses of 12.2 to 89.4 kDa (**Supplementary Table S1**). The theoretical isoelectric points (pI) varied from 5.07 to 9.79. The instability index values ranged from 29.19 to 58.46, the aliphatic index from 68.22 to 96.31, and the grand average of hydropathicity (GRAVY) values from –0.57 to 0.06. These results suggested that most StFAD proteins are hydrophilic and exhibit relatively favorable thermal stability.

Subcellular localization prediction indicated that StFAD proteins are distributed across a variety of cellular components, including the plasma membrane, endoplasmic reticulum (ER), cytoplasm, chloroplasts, mitochondria, and peroxisomes. This predicted distribution pattern may reflect subcellular compartment-dependent functional divergence. For example, StFAD1 and StFAD38 were predicted to localize to peroxisomes; StFAD2, StFAD7, StFAD9–12, StFAD26–28, StFAD32, StFAD34, StFAD37, and StFAD41–47 to the plasma membrane; StFAD3, StFAD4, StFAD14–18, StFAD20, StFAD22, StFAD23, StFAD25, StFAD30, and StFAD31 to chloroplasts; StFAD5, StFAD6, StFAD19, StFAD21, StFAD29, and StFAD40 to the cytoplasm; StFAD8 to both mitochondria and chloroplasts; and StFAD13, StFAD24, StFAD33, StFAD35, StFAD36, and StFAD39 to the ER.

### 2.2 *StFAD* genes are unevenly distributed across chromosomes

**Figure 1:**
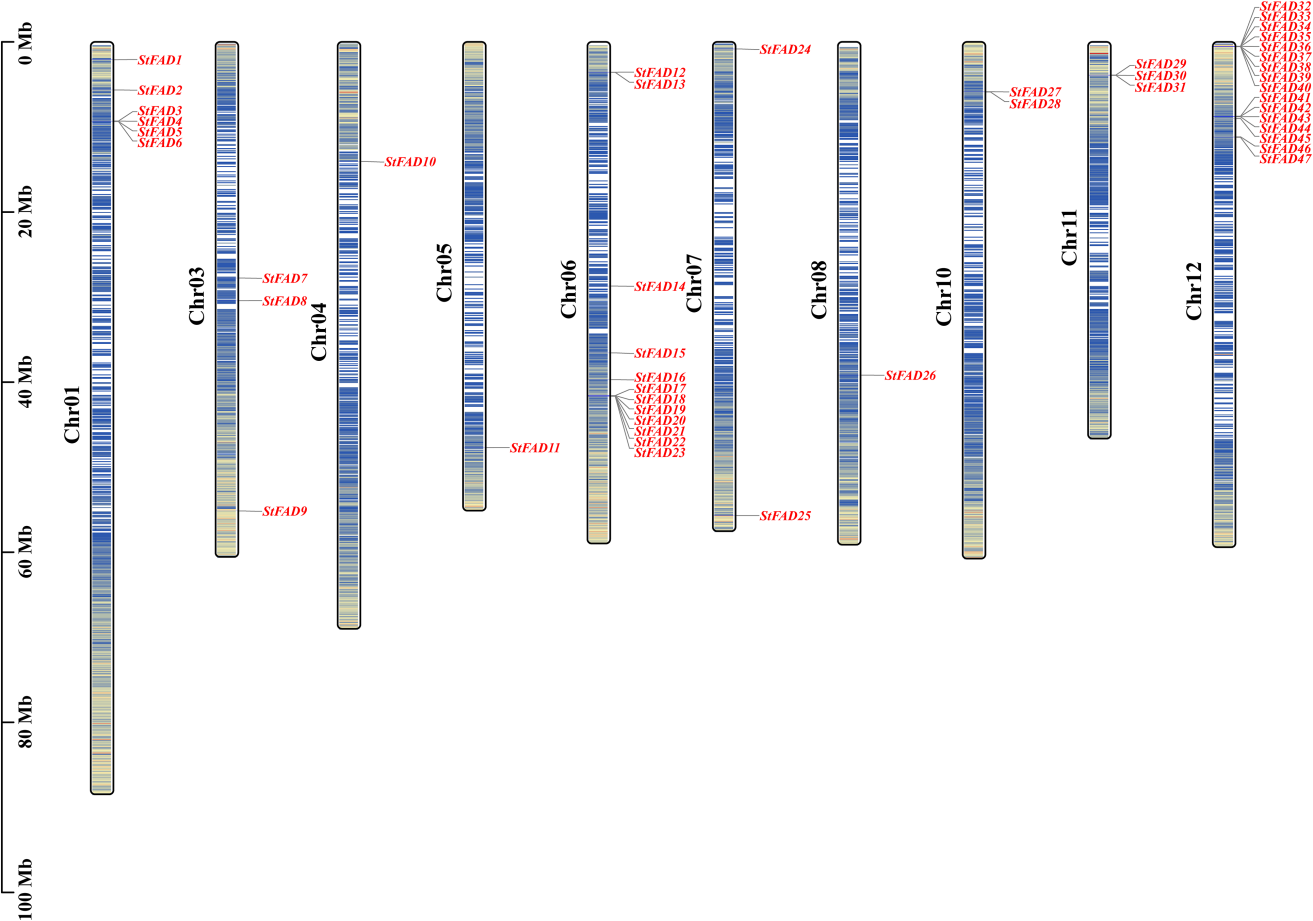
Chromosomal distribution of the *StFAD* genes in *Solanum tuberosum*. The physical locations of the 47 *StFAD* family members are mapped onto 12 chromosomes (Chr01–Chr12) based on the *S. tuberosum* genome assembly. Gene names are indicated in red and positioned according to their relative physical coordinates (Mb scale is shown on the left). The chromosome lengths are represented proportionally, with darker blue in the vertical bars along each chromosome indicating regions of greater gene density.

Chromosomal mapping revealed that the 47 *StFAD* genes were unevenly distributed across all 12 chromosomes of the *S. tuberosum* genome (**Figure 1**). Chromosome 12 contained the largest number of *StFAD* genes, with 16 members (*StFAD32*–*StFAD47*) clustered in this region, potentially attributable to gene duplication or tandem organization (**Supplementary Table S2**). The second largest cluster, containing 12 genes (*StFAD12*–*StFAD23*), was found on chromosome 6, with the genes positioned in close proximity (**Supplementary Table S3**).

Chromosome 1 harbored six genes (*StFAD1*–*StFAD6*), whereas chromosomes 4, 5, and 8 each contained only one gene (*StFAD10*, *StFAD11*, and *StFAD25*, respectively). Other genes were distributed across chromosomes 3 and 11, including *StFAD7*–*StFAD9* and *StFAD29*–*StFAD31*. This uneven genomic distribution may reflect the localized expansion of certain gene clusters.

### 2.3 Phylogenetic analysis revealed subfamily divergence among *StFAD* genes

**Figure 2:**
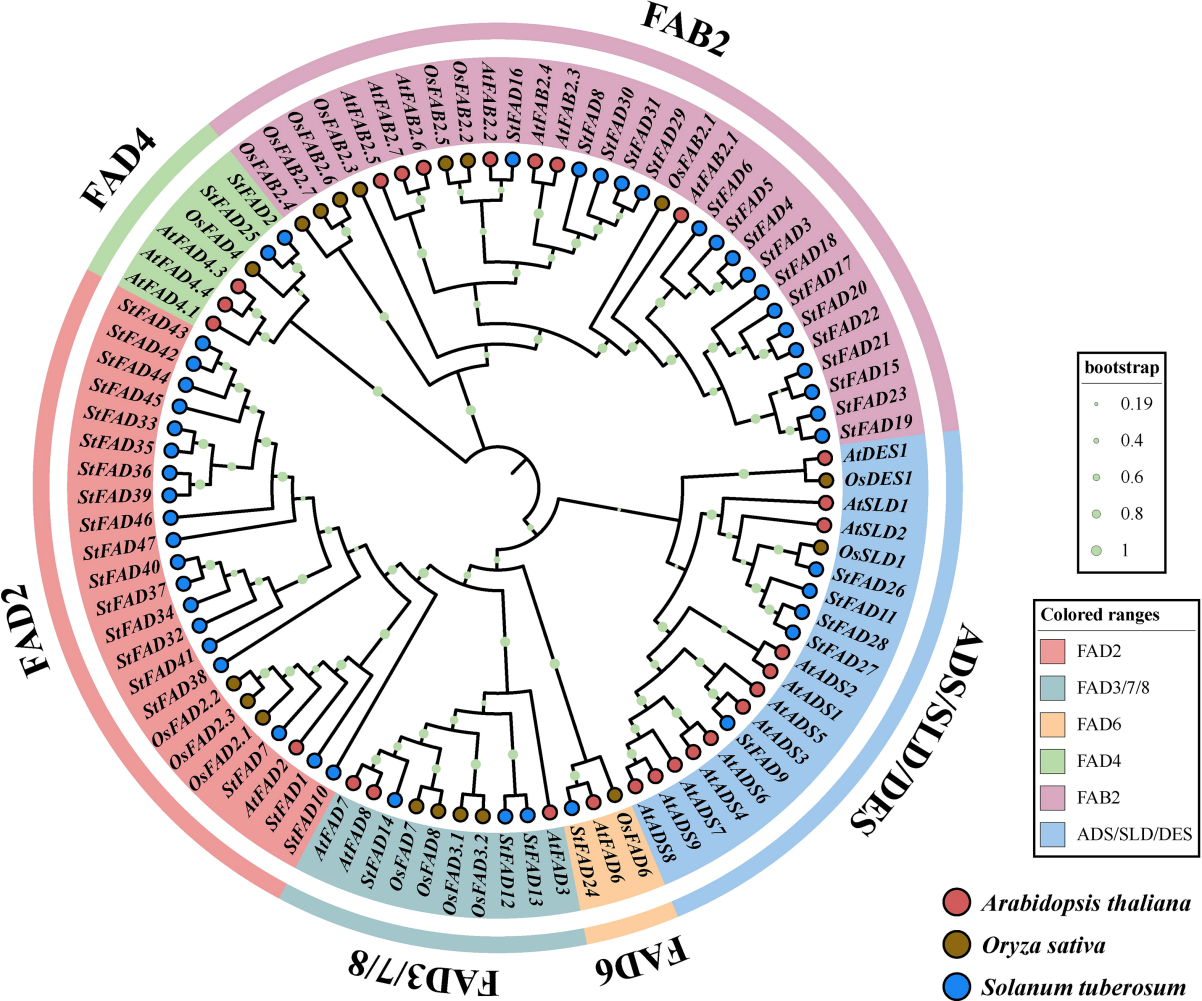
Phylogenetic analysis of *FAD* gene family members from *Solanum tuberosum*, *Arabidopsis thaliana*, and *Oryza sativa*. The maximum likelihood (ML) tree was constructed based on full-length amino acid sequences of *FAD* proteins from the three species, with branch supports indicated by bootstrap values (*n* = 1000; color-coded circles at nodes represent different bootstrap ranges as shown in the legend). *S*. *tuberosum*, *A*. *thaliana*, and *O*. *sativa* sequences are denoted by blue, red, and brown circles, respectively. The tree resolves FAD proteins into six distinct clades, FAD2, FAD3/7/8, FAD6, FAD4, FAB2, and ADS/SLD/DES, differentiated by background color.

A phylogenetic tree constructed using FAD proteins from *S*. *tuberosum*, *A. thaliana*, and *O. sativa* revealed that the *StFAD* gene family could be grouped into six major subfamilies: FAD2, FAB2, FAD3/7/8, FAD4, FAD6, and ADS/SLD/DES (**Figure 2**).

The FAD2 subfamily included 19 members from *S. tuberosum*, 6 from *A. thaliana*, and 3 from *O. sativa*. The FAB2 subfamily contained 17 members from *S. tuberosum* and 7 from *A. thaliana*, but none from *O. sativa*. The FAD3/7/8 group consisted of 3 genes each from *S. tuberosum*, *A. thaliana*, and *O. sativa*, potentially reflecting a conserved ω-3 desaturase clade across monocots and dicots. The FAD6 subfamily contained a single representative from each of the three species. The FAD4 subfamily included 2 genes from *S. tuberosum*, 3 from *A. thaliana*, and 1 from *O. sativa*. Finally, the ADS/SLD/DES group comprised 5 members from *S. tuberosum*, 12 from *A. thaliana*, and 3 from *O. sativa*. In summary, all three species shared the same six subfamilies, although differences in gene copy number within each group may reflect lineage-specific variation or possible expansion, which requires further investigation.

### 2.4 Conserved motif, domain, and gene structure analyses revealed functional divergence among StFAD proteins

**Figure 3:**
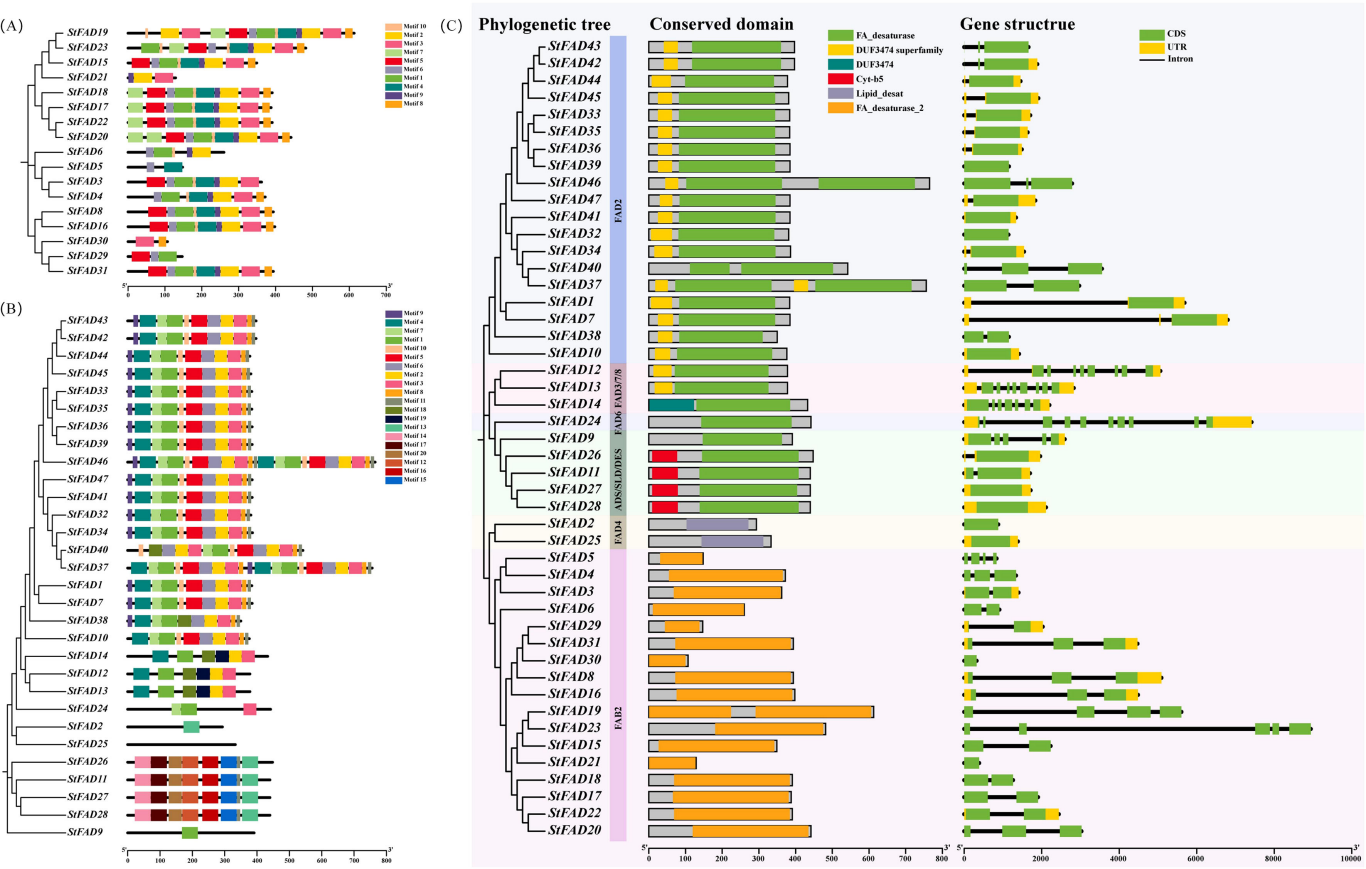
Phylogenetic relationships, conserved motif composition, domain organization, and gene structure of *StFAD* family members in *Solanum tuberosum*. **(A, B)** Distribution of conserved motifs among StFAD proteins as identified by MEME analysis. Each motif is represented by a distinct colored box, with its position corresponding to the relative location within the protein sequence. The phylogenetic trees on the left were constructed based on the full-length amino acid sequences, grouping StFAD members into different clades. **(C)** Integrated analysis of phylogenetic classification (left), conserved domain architecture (middle), and exon–intron structure (right) of *StFAD* genes. Conserved domains were annotated using Pfam and SMART, with color-coded boxes representing distinct domain types. Gene structure diagrams display coding sequences (CDS, green), untranslated regions (UTRs, yellow), and introns (black lines). Background shading denotes distinct subfamilies based on phylogenetic clustering.

To characterize the structural features of the StFAD proteins, we analyzed their conserved motifs, functional domains, and associated gene structures. Because of their distinct sequence features, motif identification in the FAD4 subfamily was performed separately from the other StFAD members to ensure accuracy. In StFAD members other than those in the FAD4 subfamily, 10 conserved motifs were identified, with motifs 1, 2, 3, 6, 9, and 10 being widely shared among most members (**Figure 3A**), suggestive of a degree of structural conservation. In contrast, StFAD21 retained only a subset of motifs, while StFAD6, StFAD29, and StFAD30 lacked several common motifs, which may indicate sequence or functional divergence (**Supplementary Table S4**).

Meanwhile, 20 motifs were identified in the FAD4 subfamily, with motifs 1–7 and 9 being the most prevalent. StFAD24 displayed a simplified motif pattern, containing only motifs 1, 3, and 7. Meanwhile, StFAD2, StFAD25–28, and StFAD9 displayed unique motif combinations.

Domain annotation further highlighted differences among subfamilies. The FA_desaturase domain was predominant among non-FAD4 family proteins, while FAD4 members uniquely harbored the Lipid_desat domain (**Figure 3B**). The Cyt-b5 domain was specific to ADS/SLD/DES members such as StFAD11, StFAD26, StFAD27, and StFAD28. Notably, DUF3474 was only identified in StFAD14 (FAD2 subfamily), which may imply a specialized functional role.

Regarding gene structure, the number of exons in *StFAD* family genes was found to range from 1 to 10 (**Figure 3C**). Genes such as *StFAD39*, *StFAD44*, and *StFAD42* had relatively simple structures with comparatively fewer exons, whereas members of the *FAD3/7/8* and *FAD6* subfamilies exhibited more complex exon–intron architectures. FAD4 members displayed the simplest structures. These structural differences may reflect subfamily-level variation and could be associated with functional divergence.

### 2.5 Intraspecies synteny and selection pressure analysis highlighted evolutionary patterns

To examine the evolutionary patterns of the *StFAD* gene family, we performed synteny relationship and selection pressure (Ka/Ks) analyses. Four syntenic gene pairs were identified: *StFAD1/StFAD33*, *StFAD8/StFAD29*, *StFAD11/StFAD27*, and *StFAD32/StFAD41* (**Supplementary Figure S1**). Among these, *StFAD1* and *StFAD33* (located on chromosomes 1 and 12, respectively) had a pairwise Ka/Ks ratio of 0.14; *StFAD8* and *StFAD29* (chromosomes 3 and 11, respectively) had a ratio of 0.26; and *StFAD11/StFAD27* and *StFAD32/StFAD41* had ratios of 0.10 and 0.22, respectively (**Supplementary Table S5**). All syntenic gene pairs exhibited Ka/Ks values of <1, indicating that they had undergone purifying selection. These findings further implied that gene duplication played a role in shaping the distribution of *StFAD* genes.

### 2.6 Cross-species synteny analysis revealed that *FAD* genes are highly conserved in solanaceae

**Figure 4:**
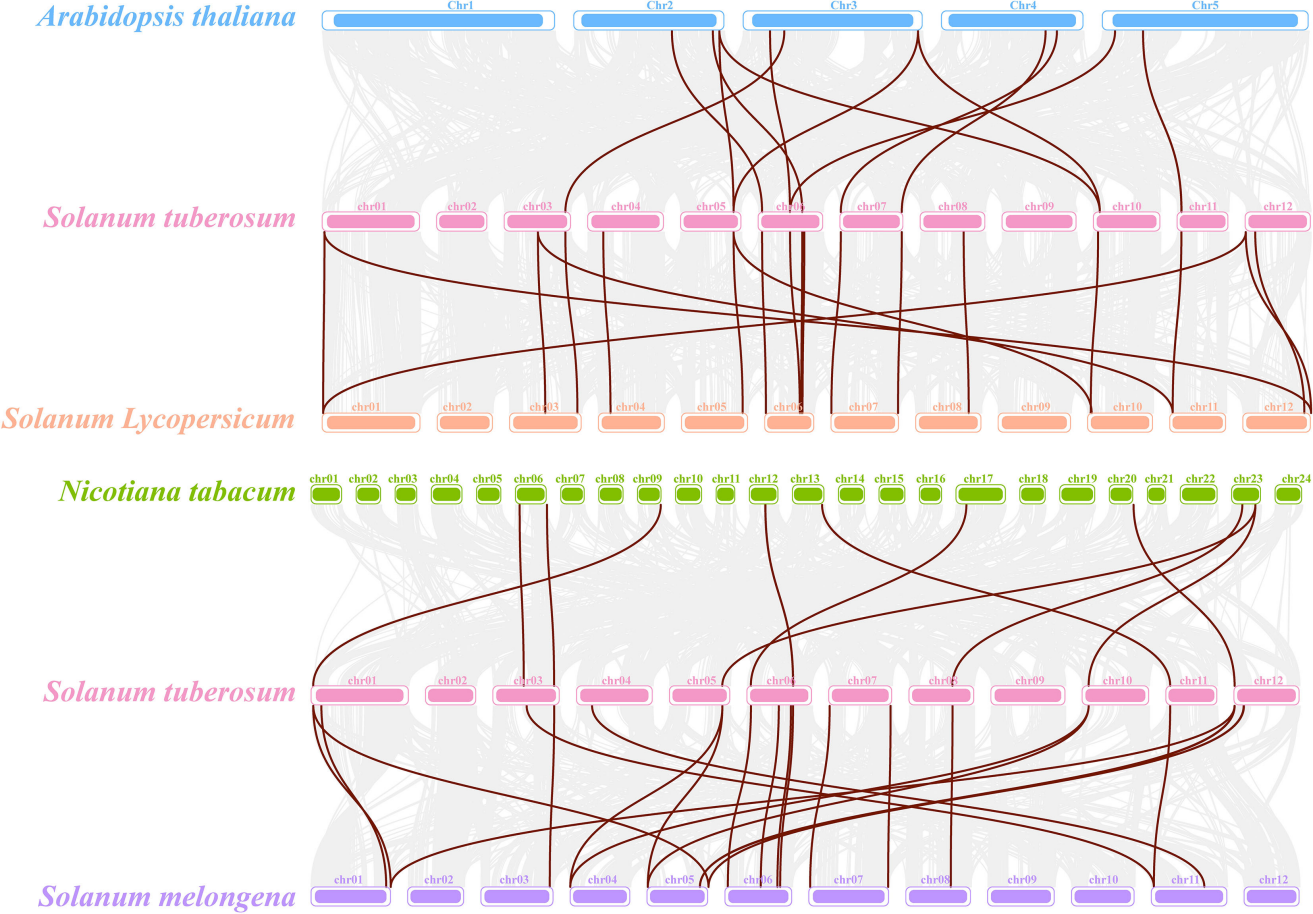
Comparative synteny analysis of *StFAD* genes in *Solanum tuberosum* and their orthologs in four plant species. The diagram illustrates syntenic blocks between *S*. *tuberosum* and each of *Arabidopsis thaliana* (blue), *S. lycopersicum* (orange), *Nicotiana tabacum* (green), and *S. melongena* (purple). Each chromosome is represented as a colored bar, with brown lines connecting orthologous gene pairs. Only collinear gene pairs involving *StFAD* family members are displayed.

A comparative synteny analysis between *S*. *tuberosum* and four representative species, *S*. *lycopersicum* (tomato), *S*. *melongena* (eggplant), *Nicotiana tabacum* (tobacco), and *A*. *thaliana*, showed varying degrees of collinearity (**Figure 4**). A total of 14 and 16 orthologous gene pairs were identified between *S*. *tuberosum* and *S*. *lycopersicum* and between *S*. *tuberosum* and *S*. *melongena*, respectively, suggesting that *FAD* genes may be relatively well conserved within the Solanaceae family. Meanwhile, fewer collinear gene pairs (9–10) were detected between *S*. *tuberosum* and *A*. *thaliana* or *N*. *tabacum*, likely reflecting evolutionary differences.

### 2.7 *Cis*-element analysis of promoter regions suggested that *StFAD* gene regulation is multifaceted

**Figure 5:**
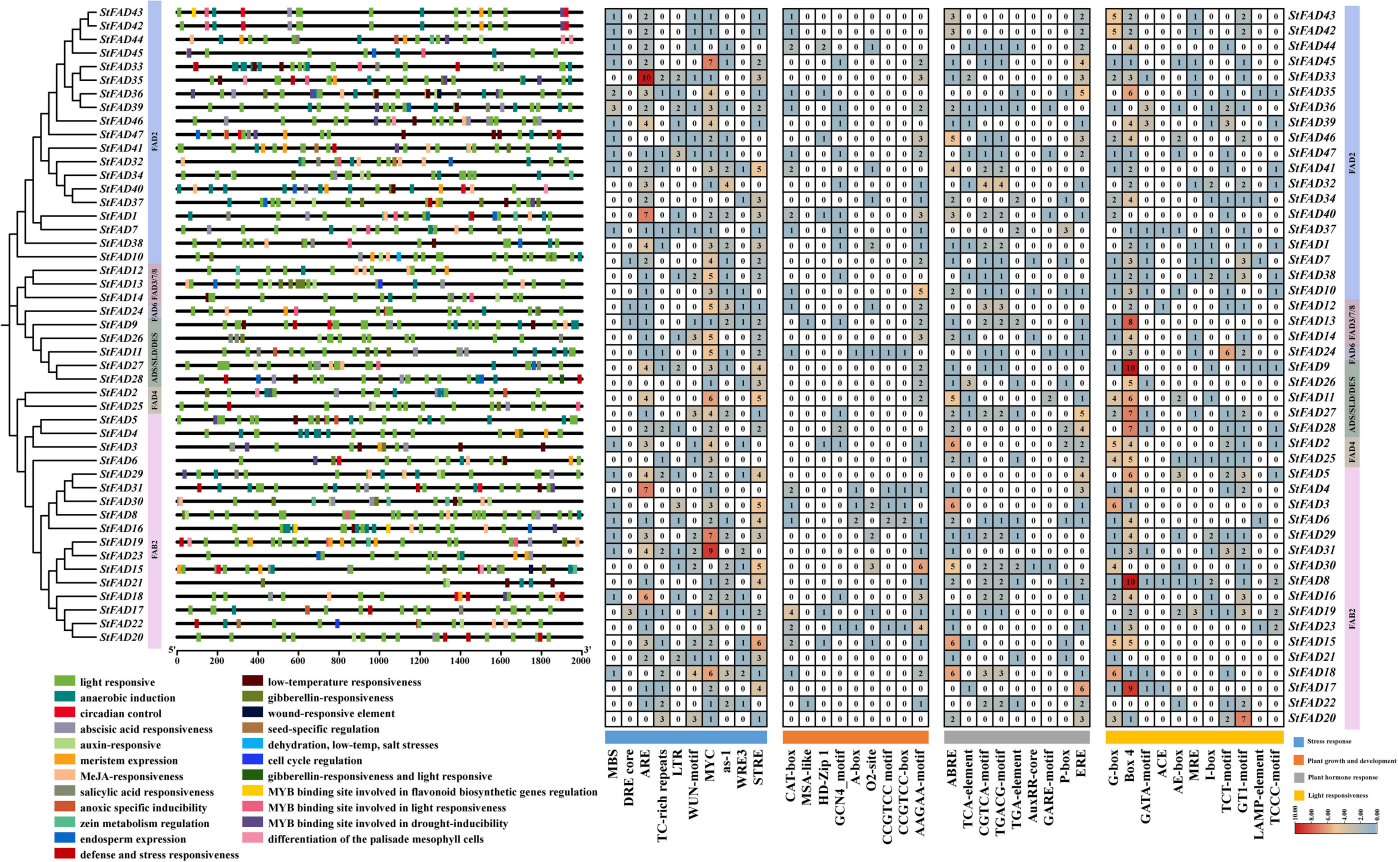
Analysis of cis-acting regulatory elements in the promoter regions of StFAD genes in Solanum tuberosum. The left panel shows the phylogenetic relationships of StFAD proteins, with promoter regions (2 kb upstream of the start codon) annotated for predicted cis-acting regulatory elements, which are color-coded by functional categories: stress response, plant growth and development, plant hormone response, and light responsiveness. The right panel shows a heatmap of the number of specific cis-elements identified in each promoter, with blue, orange, and red gradients reflecting low, moderate, and high frequencies, respectively.

To explore the transcriptional regulation of the *StFAD* genes, we analyzed the *cis*-acting elements within the 2-kb upstream promoter regions of all 47 members (**Figure 5**). The results revealed a wide range of regulatory elements related to stress responses, hormone signaling, developmental processes, and light responsiveness. Notably, *StFAD8* contained the greatest diversity of *cis*-elements (20 types), suggestive of a comparatively greater degree of regulatory complexity (**Supplementary Table S6**).

Among stress-responsive elements, ARE, MYC-binding elements, and STRE were the most frequently occurring (330 total), with higher counts observed in the promoters of *StFAD33* (FAD2 subfamily), *StFAD29*, and *StFAD31* (both FAB2 subfamily). MYC-binding elements were present in nearly all the promoters, with *StFAD31* containing nine copies. LTR elements, often associated with cold response, were detected in *StFAD3* and *StFAD47* (**Supplementary Table S7**).

Regarding developmental regulation, the AAGAA-motif and CAT-box were the most commonly detected *cis*-acting regulatory elements (92 total), with the highest frequencies observed in *StFAD2* (FAD4 subfamily) and *StFAD46* (FAD2 subfamily). Hormone-responsive elements, including ABRE, ERE, CGTCA-motif, and TGACG-motif, were also widely distributed in the promoters of StFAD genes (155 total). Light-responsive elements, particularly G-box and Box4, were similarly abundant (243 total), with *StFAD8*, *StFAD15*, *StFAD17* (FAB2 subfamily), and *StFAD9* (ADS/SLD/DES subfamily) showing the greatest enrichment. These observations indicated that *StFAD* promoters harbor diverse regulatory elements potentially associated with environmental and developmental processes.

### 2.8 Expression profiles of *StFAD* genes under cold stress and subcellular localization of *StFAD7*

#### 2.8.1 Expression profiling of *StFAD* genes across tissues and under cold stress

**Figure 6:**
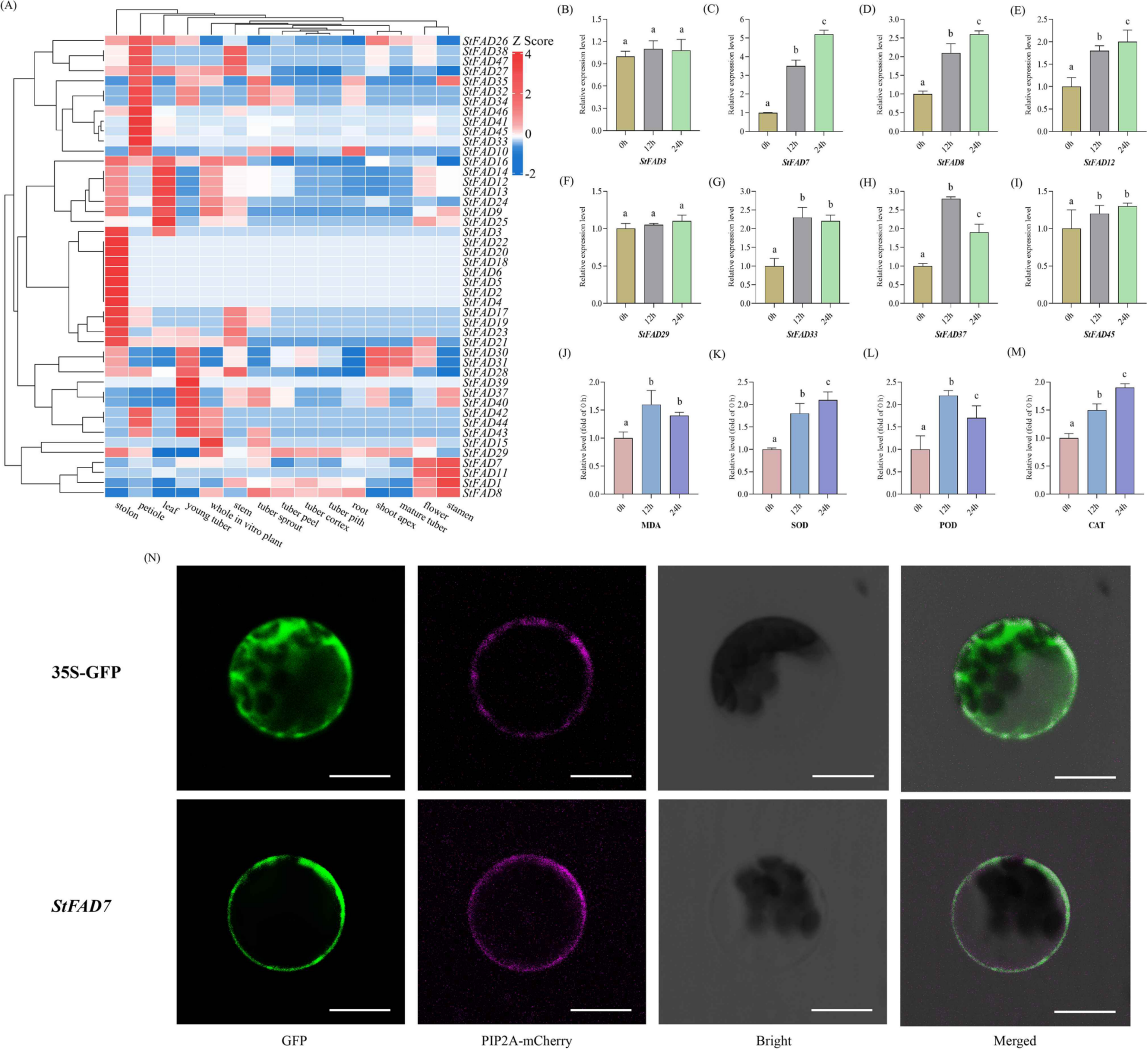
Expression profiles, physiological responses, and subcellular localization of *StFAD* genes under cold stress. **(A)** Heatmap showing the expression profiles of 47 *StFAD* genes across 15 tissues in *Solanum tuberosum*. Expression values were obtained from RNA-seq datasets and normalized using Z-score transformation for each gene. Red and blue represent relatively high and low transcript levels, respectively. **(B–I)** Relative expression levels of eight representative *StFAD* genes in potato tubers subjected to cold stress (4°C) for 0, 12, and 24 h. The X-axis represents Time after 4°C treatment (h), corresponding to 0 h (control), 12 h (early response), and 24 h (sustained/recovery phase). Gene expression was quantified by qRT-PCR and normalized to the expression level at 0 h (set as 1.00). Bars represent mean ± SD of three biological replicates. Different letters indicate significant differences between time points at p < 0.05 (Duncan’s multiple range test). **(J–M)** Changes in four physiological parameters, including MDA, SOD, POD, and CAT levels, measured in the same samples under 4°C treatment for 0, 12, and 24 h Values are expressed as relative fold of control (0 h = 1.00). Bars indicate mean ± SD (n = 3). Different letters denote statistically significant differences (p < 0.05) according to Duncan’s test. **(N)** Subcellular localization of *StFAD7* in *Nicotiana benthamiana* epidermal cells. Confocal laser scanning microscopy images show GFP fluorescence (green), the plasma membrane marker PIP2A-mCherry (magenta), bright-field images, and merged channels. The control construct (35S::GFP) exhibited ubiquitous GFP fluorescence in the cytoplasm and nucleus, whereas *StFAD7*-GFP fusion protein fluorescence was predominantly detected at the cell periphery, co-localizing with PIP2A-mCherry. Scale bars = 20 μm.

To investigate the expression dynamics of the *StFAD* family, the expression patterns of 47 *StFAD* genes were examined across 15 tissues based on RNA-seq datasets retrieved from the EnsemblPlants database. Substantial variation in transcript abundance was observed, with several genes exhibiting clear tissue-preferential expression profiles (**Figure 6A**, **Supplementary Table S8**). *StFAD1*, *StFAD7*, *StFAD8*, *StFAD12*, and *StFAD13* exhibited relatively high expression in multiple tissues, while *StFAD2*, *StFAD4*, *StFAD5*, and *StFAD41* showed low transcript abundance across most organs. Notably, *StFAD1* had the highest transcript levels in flowers and stamens (TPM = 2,691 and 5,944, respectively), while *StFAD12* and *StFAD13* exhibited elevated expression in leaves and stolons (TPM = 448 and 201, respectively). Conversely, *StFAD2*, *StFAD3*, *StFAD4*, and *StFAD5* had very low expression across most tissues, with *StFAD4* and *StFAD5* being weakly detected in stolons (TPM = 0.9). Additionally, *StFAD12*, *StFAD14*, and *StFAD24* displayed higher expression in green vegetative tissues, including leaves, petioles, and stems. These data indicate that *StFAD* genes show distinct tissue-specific and functional diversification patterns in potato.

Based on the heatmap results, the presence of stress-related cis-elements (e.g., LTR, MYC, and ABRE), and their representation across different subfamilies, eight representative genes were selected for quantitative qRT–PCR analysis: *StFAD3*, *StFAD7*, *StFAD8*, *StFAD12*, *StFAD29*, *StFAD33*, *StFAD37*, and *StFAD45*. These genes represented diverse characteristics: *StFAD7* and *StFAD8* (FAD3/7/8 subfamily) are ω-3 desaturases, potentially responsive to cold; *StFAD12* (FAD2/FAB2 clade) is highly expressed in green tissues; *StFAD33* and *StFAD37* are enriched in stress-related and developmental promoter elements; *StFAD45* is specifically expressed in petioles; and *StFAD3* carries LTR elements but has low basal transcription (**Figure 5**).

Under 4 °C treatment (0 h, 12 h, 24 h), most *StFAD* genes showed variable induction patterns (**Figures 6B–I**). *StFAD7* and *StFAD8* transcripts increased significantly at 12 h and remained elevated at 24 h, while *StFAD33* and *StFAD37* exhibited moderate induction. *StFAD12* and *StFAD45* displayed transient upregulation at 12 h, whereas *StFAD3* and *StFAD29* maintained consistently low expression across all time points. These findings suggest that only a subset of *StFAD* genes respond transcriptionally to cold stress, with *StFAD7* showing the most sustained induction.

#### 2.8.2 Physiological and biochemical responses of potato tubers to cold stress

To determine whether transcriptional changes were accompanied by physiological adjustments, four indices associated with membrane lipid peroxidation and antioxidant defense were quantified in potato tubers under cold treatment (0, 12, and 24 h at 4°C; 0 h = 1.00) (**Figures 6J–M**). MDA content increased sharply at 12 h, indicating the onset of membrane lipid peroxidation during the early chilling phase, but declined by 24 h, suggesting partial recovery and mitigation of oxidative damage. POD activity displayed a transient surge, peaking at 12 h and decreasing thereafter, whereas SOD and Catalase (CAT) activities increased progressively throughout the 24 h period, reflecting a sustained activation of ROS-scavenging systems. Collectively, these physiological trends depict a typical biphasic stress response, in which an initial oxidative burst is followed by gradual adaptation through enhanced antioxidant enzyme activity.

When compared with the transcriptional dynamics, the sustained upregulation of *StFAD7* from 12 to 24 h coincided with the attenuation of MDA accumulation and the continued rise in SOD and CAT activities. This temporal concordance suggests that *StFAD7* induction may be functionally associated with the stabilization of membrane lipids and the enhancement of antioxidant capacity during cold adaptation. Specifically, the elevated *StFAD7* expression coinciding with decreased lipid peroxidation and reinforced enzymatic ROS detoxification implies that this gene could participate in maintaining membrane homeostasis and mitigating oxidative stress through lipid desaturation processes under chilling conditions.

#### 2.8.3 StFAD7 localized to the plasma membrane

Bioinformatic analysis identified *StFAD7* as a member of the FAD3/7/8 ω-3 desaturase subfamily, with its promoter region enriched in cold- and stress-responsive cis-elements such as LTR and ARE. In addition to these regulatory features, *StFAD7* exhibited one of the strongest and most sustained transcriptional inductions under cold stress (12–24 h), coinciding with reduced MDA accumulation and enhanced SOD and CAT activities in tubers. These findings suggested that *StFAD7* may play a role in membrane lipid remodeling and oxidative stress mitigation during chilling, prompting further investigation of its subcellular localization. The gene met several additional predefined criteria, including relatively high transcript abundance across multiple tissues (average TPM = 275.3), an instability index of 35.93 (below the stability threshold of 40), and consistent bioinformatic predictions of membrane association.

To validate its localization, the full-length *StFAD7* coding sequence (without the stop codon) was fused with GFP and transiently expressed in *Nicotiana benthamiana* epidermal cells. Confocal microscopy revealed that the fluorescence signals of the *StFAD7*–GFP fusion protein were predominantly confined to the plasma membrane and overlapped with those of the plasma membrane marker PIP2A–mCherry (**Figure 6N**), consistent with in silico predictions.

Integrating these results forms a coherent line of evidence from genomic prediction to physiological validation: *StFAD7* carries multiple cold- and stress-responsive regulatory elements, is transcriptionally induced during the 12–24 h chilling phase, and displays expression dynamics consistent with decreased lipid peroxidation and elevated antioxidant activity. Its plasma membrane localization further supports a potential role in membrane lipid desaturation and ROS homeostasis under cold stress. Collectively, these findings identify *StFAD7* as a key candidate gene mediating lipid-based adaptation to chilling in *Solanum tuberosum*.

## 3 Discussion

Fatty acid desaturases comprise a family of enzymes that catalyze the formation of double bonds in fatty acid chains, thereby regulating membrane fluidity, lipid metabolism, and stress adaptability in plants (Jin et al., 2024). Their roles in maintaining cellular homeostasis under abiotic stresses, particularly cold, have been extensively documented in multiple species (Li et al., 2024; Lu et al., 2025). In this study, we systematically characterized the *StFAD* gene family in potato, offering insights into their structural features, evolutionary patterns, and potential functions in cold stress adaptation.

Our genome-wide survey revealed that the potato *FAD* gene family comprises 47 members, exhibiting an uneven chromosomal distribution with notable clusters on chromosomes 6 and 12. This pattern suggests that tandem and segmental duplications may have driven their local expansion. Comparable expansions have been documented in other crops. In soybean, 30 *GmFAD* genes have been identified and classified into seven subfamilies, with segmental duplication serving as the primary driver of expansion (Li et al., 2025). In tomato, 26 *SlFAD* genes were unevenly mapped across 10 chromosomes, forming six clades, and their promoter elements were enriched for hormone- and stress-responsive motifs (Xi et al., 2023). These lineage-specific expansions across species highlight a general evolutionary trend in which duplication events increase both family size and regulatory complexity. The expansion observed in *S. tuberosum* may reflect adaptive genome evolution in response to ecological and physiological demands.

Comparative structure–function analysis classified the 47 *StFAD* genes into six canonical subfamilies, *FAB2*, *FAD2*, *FAD3/7/8*, *FAD4*, *FAD6*, and *ADS/SLD/DES*, consistent with observations in model plants. While core motifs and domain architectures were largely conserved within subfamilies, subgroup-specific differences in motif retention and exon–intron structures were evident, indicative of potential functional specialization. Similar structural conservation combined with divergence has been reported in rice, where 20 *OsFAD* genes were identified and grouped into six subfamilies. Tandem and segmental duplications underpinned their expansion while domain conservation preserved the defining characteristics of each subfamily (Chen et al., 2019). In cucumber (*Cucumis sativus*), 23 *CsFAD* genes were identified, all retaining the characteristic histidine motifs (HXXXH, HXXHH, HXXHH) responsible for desaturase activity, and many membrane-bound members contained organelle-targeting signals (Dong et al., 2016). Similarly, in upland cotton (*G. hirsutum*), 39 *GhFAD* genes were identified, with phylogenetic clustering revealing four conserved subfamilies in which gene structures were strongly preserved. Segmental duplication again appeared to be the predominant process underlying their expansion (Feng et al., 2017). These observations indicate that *FAD* family architecture is broadly conserved across species, with lineage-specific duplications and motif variations contributing to functional divergence. Our analysis of *StFAD* genes fits this paradigm, with conservation supporting essential roles in fatty acid metabolism, and divergence contributing to regulatory and functional diversity.

Cold-responsiveness is a common feature of plant *FAD* genes, and our results confirmed that several *StFAD* members in potato exhibit transcriptional activation under cold conditions. Among them, *StFAD7* displayed the most pronounced and sustained induction, with transcript levels peaking at 12–24 h of 4°C treatment. This expression pattern coincided temporally with a transient rise and subsequent attenuation of malondialdehyde (MDA) content, as well as a continuous increase in superoxide dismutase (SOD) and catalase (CAT) activities in tubers. The synchronous upregulation of *StFAD7* and enhancement of antioxidant enzyme activity suggest that *StFAD7* induction may be functionally linked to the mitigation of oxidative stress and maintenance of membrane stability during chilling. Similar patterns have been reported in other species, where cold-induced desaturases, contribute to increased unsaturated fatty acid levels and improved tolerance to low temperatures. For instance, the overexpression of *GmFAD3A* in rice significantly improved cold tolerance at 15°C, enhancing seed germination, survival, and antioxidant enzyme activity, while concomitantly mitigating oxidative damage (Wang et al., 2019). In *A. thaliana*, *ACYL-LIPID DESATURASE 2 (ADS2)* is indispensable for cold acclimation. *ads2* mutants show increased levels of saturated fatty acids and exhibit dwarfism, sterility, and severe cold sensitivity, while *ADS2* expression is rapidly induced during chilling (Chen and Thelen, 2013). These cross-species parallels highlight the central role played by desaturases, particularly ω-3 types, in mediating cold adaptation.

The regulatory architecture of *FAD* genes further emphasizes their multifaceted roles in environmental adaptation. Our promoter analysis revealed that many *StFAD* genes harbor cis-elements that are responsive not only to cold but also to hormones (e.g., ABRE, ERE) and light (e.g., G-box, Box4). Promoter–reporter studies in *A. thaliana* demonstrated that *AtFAD7* and *AtFAD8* are differentially regulated via combinations of WRKY and MYB binding sites, along with ABA-responsive elements (Sahoo et al., 2023). Stress-responsive elements such as ABRE and DRE/CRT are well established as key regulators of gene activation under cold, drought, and salinity (Luján et al., 2021). In olive (*Olea europaea*), cold treatment during fruit development markedly upregulated *FAD7* and *FAD2.2* expression, which correlated with increases in unsaturated lipid content. This suggests that cis-element-mediated regulation may be functionally linked to membrane adaptation (Yamaguchi-Shinozaki and Shinozaki, 1994). Consistent with these findings, our analysis revealed that *StFAD7* possesses a promoter enriched in cold- and hormone-responsive cis-elements such as LTR, ARE, and ABRE, implying transcriptional regulation through multiple signaling pathways. Experimentally, *StFAD7* was confirmed to localize to the plasma membrane through GFP-fusion transient expression in *Nicotiana benthamiana* epidermal cells, where fluorescence signals overlapped with the plasma membrane marker PIP2A–mCherry. This localization agrees with bioinformatic predictions and is biologically meaningful, as the plasma membrane is the primary site of cold-induced damage. Membrane-bound ω-3 desaturases such as *StFAD7* may modulate lipid desaturation and preserve bilayer fluidity, thereby reducing mechanical rigidity and oxidative damage under chilling stress (Matteucci et al., 2011; Liu et al., 2020). Similar mechanisms have also been observed in other crops such as *Arabidopsis*, rice, and soybean, where ω-3 desaturases play conserved roles in maintaining membrane fluidity and enhancing cold stress tolerance, further supporting the conclusions of this study. Together, these molecular and cellular findings align with the observed physiological responses, reinforcing the hypothesis that *StFAD7* might contribute to cold adaptation in potato by maintaining membrane stability and facilitating lipid remodeling under low-temperature conditions. The current study integrates transcriptional induction, physiological responses, and membrane-associated localization to support the functional relevance of *StFAD7*, direct measurement of fatty acid desaturation or membrane lipid composition would provide additional biochemical confirmation. Lipidomic profiling will be incorporated in future work to determine whether cold-induced *StFAD7* activation leads to measurable changes in unsaturated fatty acid levels.

The cold-response validation in this study was performed using the commercial cultivar ‘Atlantic’ under a standard chilling treatment (4°C). ‘Atlantic’ was selected because of its agricultural relevance and its clear, reproducible sensitivity to low temperature, which facilitates correlation between transcriptional and physiological responses. Future research will systematically evaluate multiple potato genotypes and extended time courses to refine the functional characterization of *StFAD7*. Taken together, this study establishes an integrative framework connecting genome-wide identification, functional prediction, physiological validation, and subcellular localization to elucidate the potential role of *StFAD7* in potato cold adaptation. Starting from a comprehensive survey of 47 *StFAD* genes, we classified them into six subfamilies and revealed conserved ω-3 desaturases enriched in cold- and hormone-responsive cis-elements. Transcriptomic and qRT–PCR analyses further identified *StFAD7* as the most strongly and persistently induced member under 4°C treatment, coinciding with the attenuation of MDA accumulation and the enhancement of SOD and CAT activities, thereby linking gene expression to physiological adaptation. Subcellular localization demonstrated that *StFAD7* resides at the plasma membrane, the first cellular interface challenged by low temperature, consistent with its putative role in regulating membrane lipid desaturation and maintaining ROS homeostasis. Together, these multi-level findings outline a coherent progression from genome-scale screening to candidate gene validation, supporting the hypothesis that *StFAD7* contributes to lipid-based defense and redox stabilization under cold stress. Future work integrating transgenic overexpression, CRISPR/Cas9-mediated knockout, and lipidomic profiling will be essential to confirm its precise function and facilitate the breeding of cold-resilient potato cultivars.

## 4 Materials and methods

### 4.1 Identification and physicochemical property prediction of potato *FAD* genes

The genome sequence and GFF annotation files of *S. tuberosum* L. (DM 1-3–516 R44) were downloaded from the Spud DB (http://spuddb.uga.edu/, accessed on 20 January 2025). The amino acid sequences of *A. thaliana FAD* genes were obtained from The *Arabidopsis* Information Resource (TAIR) (https://www.arabidopsis.org/, accessed on 25 January 2025) and used as queries for BLASTp searches. Hidden Markov Model (HMM) profiles corresponding to the *FAD* domains, FA_desaturase (PF00487), FA_desaturase_2 (PF03405), and Lipid_desat (PF10520), were retrieved from the Pfam database (https://pfam.xfam.org/, accessed on 25 January 2025). The *FAD* genes in the potato genome were identified using HMMER 3.0 (http://hmmer.org/, accessed on 30 January 2025) by searching for sequences containing these domains. The presence of conserved FAD-related domains in candidate genes was confirmed through Pfam, SMART, and InterProScan. For genes with multiple transcripts, the longest coding sequence (CDS) isoform was selected as representative.

The physicochemical properties of the predicted StFAD proteins, including amino acid length, pI, molecular mass, instability index, aliphatic index, and GRAVY, were calculated using the ProtParam tool (https://web.expasy.org/protparam/, accessed on 5 February 2025). Subcellular localization was predicted using the WoLF PSORT server (https://wolfpsort.hgc.jp/, accessed on 8 February 2025), and the prediction with the highest score was retained. Chromosomal location information was extracted from the genome annotation file and visualized using the “Visualize Gene Location from GTF/GFF” module in TBtools.

### 4.2 Phylogenetic, gene structure, and conserved motif analyses of *StFAD* genes

The amino acid sequences for the *FAD* genes of *S. tuberosum*, *A*. *thaliana*, and *O. sativa* were aligned using ClustalW. A phylogenetic tree was constructed in MEGA 12 using the Maximum Likelihood method with 1,000 bootstrap replicates. The tree was visualized and edited using iTOL (https://itol.embl.de/, accessed on 10 February 2025).

Conserved motif structures of the StFAD proteins were identified using the MEME Suite (http://meme-suite.org/, accessed on 10 February 2025). For the FAB subfamily, the maximum number of motifs was set to 10, with a minimum motif width of 6 and a maximum width of 50. For other subfamilies, the maximum number of motifs was set to 20, with the same width parameter. Gene structures, including CDS and untranslated regions (UTRs), were extracted from the genome annotation files and visualized using TBtools v2.225.

### 4.3 Synteny analysis and gene duplication of *StFAD* genes

BLASTp searches (E-value < 1e−10) were performed to identify homologous *FAD* genes in *S*. *tuberosum*. Syntenic relationships among *StFAD* genes were analyzed using MCScanX with default parameters, and collinear blocks and duplicated gene pairs were visualized in TBtools.

For interspecies synteny analysis, genome sequences and annotation files for *A*. *thaliana* were obtained from Phytozome v13 (https://phytozome-next.jgi.doe.gov/, accessed on 13 February 2025), those for tomato (*S. lycopersicum*) were obtained from NCBI (https://www.ncbi.nlm.nih.gov/, accessed on 13 February 2025), those for tobacco (*N. tabacum*) were derived from the Sol Genomics Network (https://solgenomics.net/, accessed on 15 February 2025), and those for eggplant (*S. melongena*) were obtained from the Eggplant Genome Database (http://www.eggplant-hq.cn/Eggplant/home/index, accessed on 16 February 2025). MCScanX was used to assess collinearity between *S*. *tuberosum* and each of these species.

### 4.4 *Cis*-Acting regulatory element analysis in *StFAD* gene promoters

For each *StFAD* gene, a 2,000-bp sequence upstream of the translation start codon (ATG) was extracted from the *S. tuberosum* genome using TBtools. *Cis*-acting regulatory elements were predicted using PlantCARE (https://bioinformatics.psb.ugent.be/webtools/plantcare/html/, accessed on 18 February 2025). The Basic Biosequence View and HeatMap modules in TBtools were used to visualize and categorize *cis*-elements associated with stress responses, plant growth and development, phytohormone responsiveness, and light responsiveness.

### 4.5 Expression profiling of *StFAD* genes

#### 4.5.1 Tissue-specific expression analysis based on RNA-seq

To investigate the tissue-specific expression patterns of the *StFAD* gene family, RNA-seq data from 15 tissues in *S*. *tuberosum*, flower, leaf, mature tuber, petiole, root, shoot apex, stamen, stem, stolon, tuber cortex, tuber peel, tuber pith, tuber sprout, whole *in vitro* plant, and young tuber, were retrieved from the EnsemblPlants database. The expression levels of 47 *StFAD* members were quantified as TPM (Transcripts Per Million). Genes with expression levels below 0.5 TPM were categorized as weakly expressed. TPM values were log_2_-transformed and visualized as heatmaps using TBtools to illustrate the spatial expression patterns of *StFAD* genes across different tissues.

#### 4.5.2 Quantitative qRT–PCR validation under cold stress

To validate cold-responsive expression patterns, quantitative RT–PCR (qRT–PCR) was performed using tubers of the cold-sensitive commercial cultivar ‘Atlantic’. Tubers were subjected to chilling stress at 4°C, and samples were collected at 0 h (control), 12 h, and 24 h. Each treatment included three biological replicates. Total RNA was extracted using the TaKaRa MiniBEST Plant RNA Extraction Kit, and first-strand cDNA was synthesized with PrimeScript™ RT Master Mix (TaKaRa) (Ye et al., 2018). Gene-specific primers were designed with Primer3 and are listed in **Supplementary Table S9**. qRT–PCR assays were conducted on an Applied Biosystems QuantStudio 6 Real-Time PCR System, with *StActin97* as the internal control. Relative expression levels were calculated using the 2^−ΔΔCt method (Livak and Schmittgen, 2001), with 0 h serving as the calibrator. Statistical analyses were performed using one-way ANOVA followed by Duncan’s multiple range test (*p* < 0.05). Results are presented as mean ± SD from three independent biological replicates, and data visualization was carried out using GraphPad Prism 9.

### 4.6 Physiological and biochemical measurements under cold stress

Tubers of the commercial cultivar ‘Atlantic’ (uniform growth, healthy) were subjected to chilling at 4 °C and sampled at 0, 12, and 24 h (n = 3 biological replicates per time point). For each time point, the tuber used for physiological assays was ID-matched to the sample used for qRT-PCR to enable expression–phenotype correlation. Data were normalized as fold of control with 0 h set to 1.00.

#### 4.6.1 Crude extract preparation and protein assay

Approximately 0.5 g of tuber tissue was homogenized in 5 mL of ice-cold extraction buffer (50 mM potassium phosphate, pH 7.8, 1 mM EDTA, 1% (w/v) PVPP, 0.1% (v/v) Triton X-100). Homogenates were centrifuged (12,000 g, 15 min, 4°C), and the supernatant was used immediately for enzyme assays. Soluble protein was quantified by the Bradford method using BSA as the standard.

#### 4.6.2 Malondialdehyde

Lipid peroxidation was estimated via thiobarbituric acid reactive substances (TBARS) with minor modifications (Heath and Packer, 1968). 0.5 mL extract was mixed with 2.0 mL of 0.5% (w/v) TBA in 10% (w/v) trichloroacetic acid (TCA), heated at 95 °C for 20 min, rapidly cooled on ice, and centrifuged (12,000 g, 10 min). Absorbance of the supernatant was read at 532 and 600 nm. MDA was calculated as:MDA (nmol mL^−1^)=(A532–A600)/155mM^−1^cm^−1^ × 10³,and expressed as nmol g⁻¹ fresh weight (FW).

#### 4.6.3 Superoxide dismutase

SOD activity was measured by inhibition of nitroblue tetrazolium (NBT) photoreduction (Beauchamp and Fridovich, 1971). Reaction mixtures (3 mL) contained 50 mM phosphate buffer (pH 7.8), 13 mM methionine, 75 µM NBT, 2 µM riboflavin, 0.1 mM EDTA, and enzyme extract. After illumination (4000 lx, 10 min), A560 was recorded; non-illuminated and no-enzyme blanks were included. One unit (U) of SOD activity was defined as the amount of enzyme causing 50% inhibition of NBT reduction. Activities were expressed as U mg⁻¹ protein.

#### 4.6.4 Peroxidase

POD activity was determined using guaiacol oxidation (Chance and Maehly, 1955). The 3 mL assay contained 50 mM phosphate buffer (pH 6.0), 20 mM guaiacol, 10 mM H_2_O_2_, and enzyme extract. The increase in absorbance at 470 nm (ϵ = 26.6 mM⁻¹ cm⁻¹ for tetraguaiacol) was recorded over 1–2 min. One unit was defined as ΔA470 = 0.01 min⁻¹ under assay conditions and expressed as U mg⁻¹ protein.

#### 4.6.5 Catalase

CAT activity was assayed by monitoring H_2_O_2_ decomposition at 240 nm (Aebi, 1984). The 3 mL reaction contained 50 mM phosphate buffer (pH 7.0), 10 mM H_2_O_2_, and enzyme extract. The decrease in A240 was recorded for 1–2 min (ϵ = 39.4 mM⁻¹ cm⁻¹). Activities were expressed as U mg⁻¹ protein.

#### 4.6.6 Statistics and visualization

Values are mean ± SD (n = 3). Data were checked for normality (Shapiro–Wilk) and homogeneity (Levene) before one-way ANOVA; group means were compared by Duncan’s multiple range test (*p* < 0.05). Plots were generated in GraphPad Prism 9.

### 4.7 Subcellular localization assays

Based on transcriptomic, promoter, and physicochemical analyses, StFAD7 was selected as a representative candidate for experimental validation due to its high expression under cold stress, enrichment of cold-responsive cis-elements, and bioinformatic prediction of membrane localization. The full-length CDS (without a stop codon) was cloned into the p16318-hGFP expression vector *via* seamless cloning. Recombinant plasmids were amplified in *Escherichia coli* DH5α, and the purified plasmid DNA was introduced into *Nicotiana benthamiana* mesophyll protoplasts using polyethylene glycol (PEG)-mediated transfection, followed by washing with W5 solution (Kobayashi et al., 2006). After incubation for 16–24 h, GFP fluorescence was observed using a laser scanning confocal microscope (excitation: 488 nm; emission: 500–550 nm). Chlorophyll autofluorescence was detected at 587 nm (Zhu et al., 2023).

## 5 Conclusions

This study provides a comprehensive overview of the FAD gene family in *Solanum tuberosum*, revealing its structural diversity and potential roles in cold stress adaptation. A total of 47 *StFAD* genes were identified and classified into six subfamilies, exhibiting conserved domain architectures but distinct exon–intron patterns, suggesting both evolutionary conservation and functional divergence. Promoter analysis highlighted the enrichment of cold, hormone, and light-responsive cis-elements, indicating complex transcriptional regulation. Expression profiling and qRT–PCR validation demonstrated that *StFAD7* is markedly induced by low-temperature treatment (4°C, 12–24 h), coinciding with reduced lipid peroxidation and enhanced antioxidant enzyme activities. Subcellular localization confirmed that StFAD7 is a membrane-associated desaturase, supporting its role in maintaining lipid fluidity and redox balance during chilling stress.

## Data Availability

The original contributions presented in the study are included in the article/**Supplementary Material**. Further inquiries can be directed to the corresponding authors.
