# Supplementary material for: Genome-wide analysis of the FAD gene family in Solanum tuberosum L. reveals its involvement in cold stress tolerance
Source: Front Plant Sci. 2025 Dec 10;16:1736660. doi: 10.3389/fpls.2025.1736660 (PMC12728355; doi:10.3389/fpls.2025.1736660)
Supplement: Supplementary file 1 [file Supplementaryfile1.docx]

Supplementary Material

# Supplementary Figures


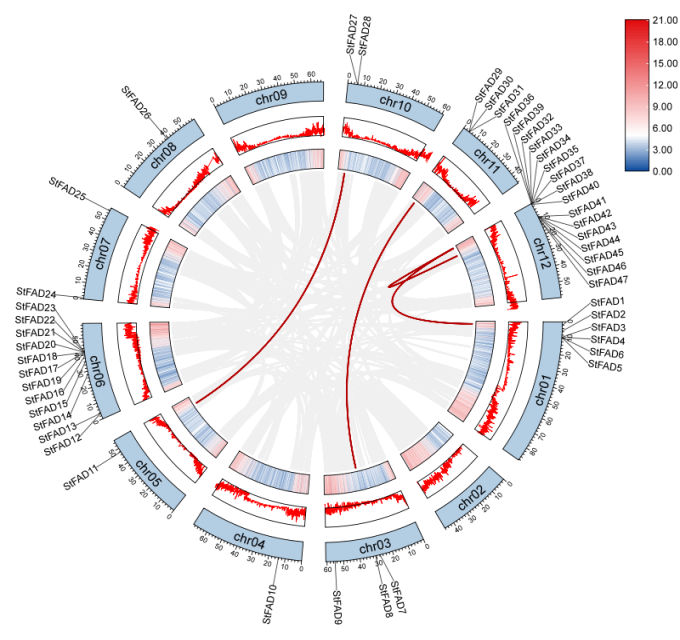


**Supplementary Figure 1.** Chromosomal distribution, syntenic relationships, and tissue-specific expression profiles of StFAD genes in Solanum tuberosum. The outermost track represents the 12 chromosomes (Chr01–Chr12) with physical positions in megabases (Mb). StFAD gene locations are marked on each chromosome. The second track (heatmap) shows the normalized expression levels (FPKM) of *StFAD* genes across various tissues, with red and blue indicating high and low expression, respectively, as indicated by the scale bar (0–21). The third track (red line plots) depicts variation in gene density along the chromosomes. Grey curves in the background denote all syntenic relationships in genome, while red curves specifically highlight segmental duplication events between StFAD genes.
